# Capsule Independent Antimicrobial Activity Induced by Nanochitosan against *Streptococcus pneumoniae*

**DOI:** 10.3390/polym13172924

**Published:** 2021-08-30

**Authors:** Fulwah Y. Alqahtani, Fadilah S. Aleanizy, Eram El Tahir, Hessa Alowais, Assalh Binkelaib, Bdour Alwathlan, Asmaa Al-Bdrawy, Anders P. Håkansson, Ibrahim Alsarra

**Affiliations:** 1 Department of Pharmaceutics, College of Pharmacy, King Saud University, Riyadh 11495, Saudi Arabia; faleanizy@ksu.edu.sa (F.S.A.); ekeltahir@ksu.edu.sa (E.E.T.); ihessa.aziz@gmail.com (H.A.); a.binkeliab@gmail.com (A.B.); bdour.z.alwathllan@gmail.com (B.A.); asmaabdrawy2@gmail.com (A.A.-B.); ialsarra@ksu.edu.sa (I.A.); 2 Department of Translational Medicine, Division of Experimental Infection Medicine, Lund University, SE-21428 Malmö, Sweden; anders_p.hakansson@med.lu.se

**Keywords:** *Streptococcus pneumoniae*, chitosan nanoparticles, nanochitosan, antimicrobial, hemolysis, capsule

## Abstract

Background: *Streptococcus pneumoniae* remains a major cause of community-acquired pneumonia, meningitis, and other diseases, contributing significantly to high morbidity and mortality worldwide. Although it responds to antibiotics, their use is becoming limited due to the rise in antibiotic resistance, which necessitates the development of new therapeutics. Nanotechnology is used to counteract antimicrobial resistance. In this regard, polymeric nanoparticles (NPs) made of natural, biodegradable, biocompatible, and cationic polymers such as Chitosan (CNPs) exhibit wide-spectrum antimicrobial activity. Therefore, this study aimed to prepare CNPs, characterize their physiochemical characteristics: particle size (PZ), polydispersity index (PDI), and zeta potential (ZP), and investigate their antimicrobial activity against *Streptococcus pneumoniae* TIGR4 (virulent serotype 4) and its capsular mutant (∆cps). Methods: CNPs were prepared at 1, 2.5, and 5 mg/mL concentrations using the ion gelation method. Then, PZ, PDI, and ZP were characterized using a Zetasizer. Transmission electron microscopy (TEM) was used to visualize the CNP’s morphology. Broth and agar dilution methods were used to assess their antimicrobial activity. Cytotoxicity of prepared NPs on A549 cells and their effect on pneumococcal hemolysis were also investigated. Results: Spherical CNPs were produced with PZ ranging from 133.3 nm ± 0.57 to 423 nm ± 12.93 PDI < 0.35, and ZP from 19 ± 0.115 to 27 ± 0.819. The prepared CNPs exhibited antibacterial activity against TIGR4 and its capsule mutant with a minimum inhibitory concentration (MIC90) of 0.5 to 2.5 mg/mL in a non-acidic environment. The hemolysis assay results revealed that CNPs reduced bacterial hemolysis in a concentration-dependent manner. Their mammalian cytotoxicity results indicated that CNPs formed from low concentrations of Chitosan (Cs) were cytocompatible. Conclusion: Nanochitosan particles showed anti-pneumococcal activity regardless of the presence of capsules. They resulted in a concentration-dependent reduction in bacterial hemolysis and were cytocompatible at a lower concentration of Cs. These findings highlight the potential of CNPs in the treatment of pneumococcal diseases.

## 1. Introduction

*Streptococcus pneumoniae* (the pneumococcus) is a gram-positive bacterium and a major human pathogen. It causes a spectrum of diseases, including bacterial otitis media, pneumonia, meningitis, and septicemia, and contributes to high morbidity and mortality globally, particularly in young children, the elderly, and immunocompromised individuals [1]. There are effective antibiotics used for the treatment of pneumococcal infections; however, their increased utilization and incomplete treatment schemes have led to the emergence of multidrug-resistant pneumococcal strains that spread worldwide [2]. According to a recent report from the World Health Organization (WHO) published in 2014 regarding global antibiotic resistance, pneumococcus was classified as one of nine bacteria of international concern [3]. Therefore, alternative antimicrobial strategies to combat *S. pneumoniae* infection are direly needed.

The use of nanotechnology, particularly polymeric nanoparticles (PNPs), has emerged in recent years as an alternative tool to develop antimicrobial therapeutics [4,5]. In this regard, NPs made from natural polymers such as Chitosan (Cs) have received much attention lately. This is owed to Cs biocompatibility, biodegradability, non-toxicity, and broad-spectrum antimicrobial activity against fungi, gram-positive, and gram-negative bacteria [6,7,8]. Chitosan is a polymer of a linear polysaccharide constituting of β -(1–4)- linked *N*-acetyl-d-glucosamine, which is generated from the partial or total deacetylation of chitin [6,9]. A recent recommendation from the United States Food and Drug Administration (USFDA) has recognized shrimp-derived Chitosan as Generally Recognized As Safe (GRAS) for general use in foods [10]. The exhibited antimicrobial properties of Cs are explained by variable mechanisms; nevertheless, the exact mechanism still needs to be elucidated [6,7,11]. The most commonly suggested mechanism is based on the electrostatic attraction between Cs and the anionic surface of bacteria leading to changes in cell membrane permeability and leakage of intracellular bacterial components and consequently cell death [6,7,11]. Nonetheless, the observed antimicrobial efficacy of Cs is influenced by many factors, including molecular weight, degree of deacetylation, and positive charge content [6,7,11].

Several reports have shown a higher antimicrobial activity of nanoparticles obtained from Cs (CNPs) against a wide range of pathogens than the activity of Cs itself [12,13,14,15,16,17,18]. This superior activity could be attributed to the small size and quantum size effect of CNPs. Considering the global rise in pneumococcal antimicrobial resistance and the potential of CNPs to act as antimicrobial agents, this study was therefore conducted to formulate nanochitosan, investigate their physicochemical properties, and evaluate their potential antimicrobial activity against Streptococcus pneumoniae.

## 2. Materials and Methods

### 2.1. Materials

Low molecular weight Chitosan (LMW, 50–190 kDa, Degree of deacetylation; DD = 75–85%), tripolyphosphate (TPP), and acetic acid were purchased from Sigma-Aldrich (St. Louis, MO, USA). Blood agar plates (BA) and Todd Hewitt broth supplemented with 0.5% yeast extract (THYB) were obtained from Merck (Darmstadt, Germany). All solvents and chemicals were of analytical grade. Dulbecco’s modified Eagle’s medium (DMEM), fetal bovine serum (FBS), and antibiotic-antimycotic solution were obtained from Gibco (Grand Island, NY, USA).

### 2.2. Preparation of CNPs

As described previously, with slight adjustment [16,19], CNPs were formulated utilizing the ionic gelation method. Cs solutions were prepared at concentrations of 1, 2.5, and 5 mg/mL in 1% *v/v* acetic acid. Then, 1 mL of 0.1% *w/v* TPP solution was added to 4 mL of Cs under continuous magnetic stirring at 800 rpm for 1 h to form the nanoparticles. Following centrifugation at 10,000× g for 20 min, recovered NPs were washed twice by centrifugation and reconstituted in distilled water and stored at 4 °C for NP characterization and further experiments.

### 2.3. Characterization of Formulated NPs

The particle size, zeta potential, and polydispersity index (PDI) were determined using dynamic light scattering with a Zetasizer (Particle Sizing Systems, Port Richey, FL, USA). All measurements were carried out in triplicate and reported as the mean ± standard deviation (SD). The NP morphology was examined using the Tecnai transmission electron microscope (TEM) (Hillsboro, OR, USA).

### 2.4. Stability Studies

The stability of prepared CNPs was evaluated at different possible proper storage temperatures (25 °C, 4 °C, and −30 °C) for 3 months. At predetermined time points (0, 40, and 80 days), samples were tested physically and characterized as a function of particle size (nm), polydispersity index, and zeta potential (mV).

### 2.5. Bacterial Strains and Growth Conditions

The pneumococcal strains and mutants used in this study are presented in Table 1. The bacteria were cultured on blood agar plates (BA) overnight or in THYB at 37 °C in 5% CO_2_ and used in the antimicrobial testing.

### 2.6. Antimicrobial Assay

In this study, broth microdilution assays were used to determine the minimal inhibitory concentration of 90 (MIC_90_) of the CNPs according to the Clinical and Laboratory Standard Institute (CLSI) protocols and as described previously [16,18,22]. The CNPs were diluted in Todd Hewitt broth (THYB) at twofold serial dilutions in standard Bioscreen C 100-well microtiter plates (Labsystems Oy, Helsinki, Finland). For inoculum preparation, all of the bacterial cell suspensions were first adjusted to McFarland 0.5 (1–2 × 108 CFU/mL). The suspension was further diluted to provide a final inoculum density of 5 × 105 CFU/mL in the wells of the microdilution plates. The plates were incubated for 7.5 h at 37 °C and 5% CO_2_, and the optical density (OD) of the bacterial growth was recorded every 15 min at 600 nm, and the MIC90 was calculated. The OD reading of each concentration of tested NPs and THYB medium alone were subtracted from the OD of the wells inoculated with bacteria to eliminate background. The MIC90 was defined as the minimal inhibitory concentration of the CNPs that reduced bacterial growth by 90%.

For the killing assay, bacteria were diluted to 9 × 106 CFU/mL in THYB in the presence of CNPs at 37 °C for 5 h. After incubation, bacteria were serially diluted and plated on BA and grown at 37 °C and 5% CO_2_ overnight, after which viable colonies were quantified. The concentrations were presented as CFUs/mL.

### 2.7. Hemolytic Activity Assay

Hemolytic activity was assayed as reported previously with minor modifications [23]. Overnight cultures of pneumococcal strains were sub-cultured in THYB until reaching an OD600 of 0.5. The culture was then diluted to an OD600 of 0.09 and co-incubated with serially diluted CNPs for 5 h. After incubation, the bacteria were pelleted by centrifugation at 12,000 rpm for 5 min (min), and supernatant from each sample was collected and filtered through a 0.22 µm syringe filter (Merck Millipore, Burlington, MA, USA).

A blood sample was collected from a healthy volunteer with immediate transfer to an anticoagulant citrate tube, centrifuged at 500× *g* for 5 min in a 15 mL tube, and the supernatant was discarded and replaced with phosphate-buffered saline (PBS) for platelet removal. This step was repeated twice to obtain purified erythrocytes in the pellet, and the pellet was resuspended in a final volume of 1.5 mL PBS. For hemolytic activity assay, 675 µL of THYB was mixed with 300 µL of bacterial supernatant and 25 µL of purified erythrocyte suspension and incubated at 37 °C for 30 min. Freshly prepared 0.1% Triton X-100 was used as a positive control (100% hemolysis), and PBS was used as negative control (0% hemolysis). The mixture was centrifuged at 10,000× *g* for 1 min, and supernatants were collected and transferred to a 96-well plate, and absorbance of each sample was measured for hemoglobin content (at 590 nm) in triplicate using an ELISA plate reader.

### 2.8. Cytotoxicity Assay

The cytotoxicity of the CNPs was investigated using alamarBlue as previously described [24]. Briefly, A549 cells (Human lung adenocarcinoma cell line CCL 185, ATCC) were seeded in 24-well plates in DMEM media supplemented with 10% FBS and 1% antibiotic-antimycotic solution overnight, until 70% to 90% confluency had been reached. The cells were then treated with the CNPs (0.016–2.5 mg/mL, for 24 and 48 h. After experimental treatment, the cell media was discarded and replaced with fresh media containing 10% alamarBlue solution, and the cells were incubated for 2–4 h at 37 °C, 5% CO_2_. Fluorescence was recorded using 550 nm excitation and 590 nm emission wavelengths in a SpectraMax M5 fluorometer (Molecular Devices, CA, USA). The viability of treated cells was expressed as a ratio of non-treated cells after subtracting the signal from media alone from both samples as follows:

Cell Viability (%) = (Fluorescence of cells treated with CNPs)/(Fluorescence of cells non treated) × 100

### 2.9. Statistical Analysis

Data were presented as the mean ± standard deviation (SD). Analysis of the data was performed using GraphPad Prism version 8.0.0 for Mac OS (GraphPad Software, Inc, CA, USA). Student’s *t*-test and ANOVA tests were used when appropriate. A *p*-value of <0.05 was considered significant.

## 3. Results

### 3.1. Characterization of Nanochitosan

In the present study, CNPs were successfully obtained adopting the ion gelation method as reported by former studies [16,19], which is based on crosslinking between cationic Cs and anionic TPP. As shown in Table 2, the dynamic light scattering results demonstrated average particle sizes of 133.3, 177, and 423 nm for CNPs prepared from Cs solution with concentrations of 1 mg/mL, 2.5 mg/mL, and 5 mg/mL, respectively. The results revealed that increasing Cs concentration led to a significant increase in the particle size and ζ-potential of the synthesized NPs (*p* < 0.05, Table 2). In this respect, NPs formulated from Cs solution of concentration 1 mg/mL (CNPs1 mg/mL) displayed the smallest particle size (133.3 nm ± 0.57) and ζ-potential (17 mV ± 0.115).

**Figure 1:**
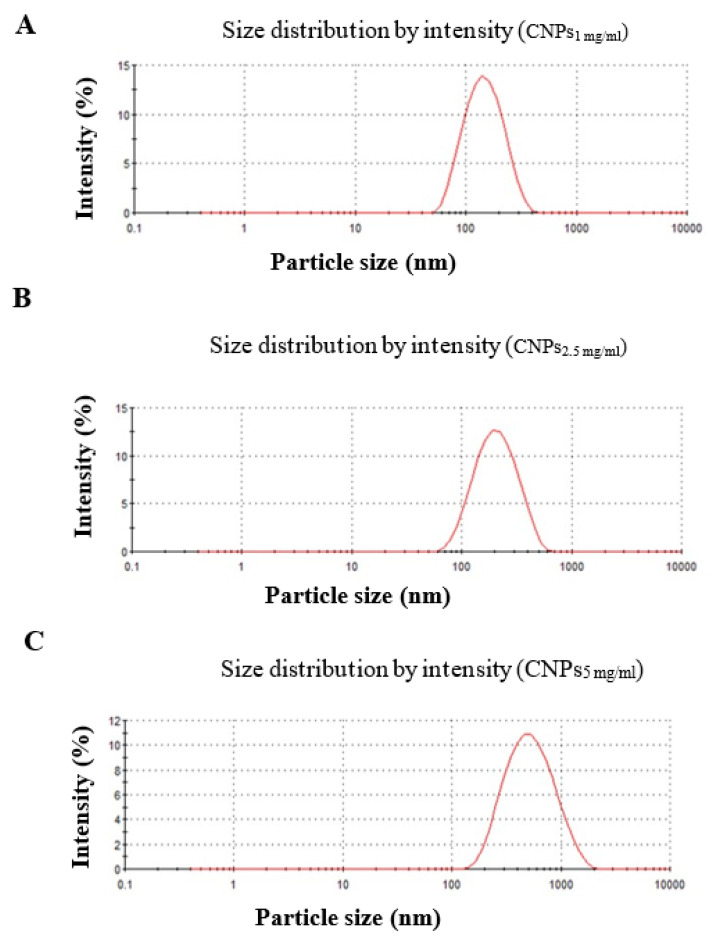
DLS spectra on of hydrodynamic size distribution of synthesized NPs.

**Figure 2:**
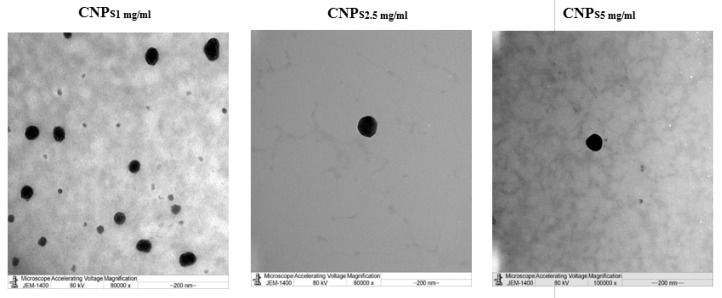
TEM images of formulated CNPs.

Consequently, the largest particle size and ζ-potential were obtained with CNPs prepared from 5 mg/mL Cs solution (CNPs_5 mg/mL_), which provided particles of sizes 423 nm ± 12.93 and ζ-potential of 27 mV ± 0.819, respectively. The PDI results featured in Table 2 indicated high homogeneity of prepared NPs, as their PDI values were ≤0.281 (Table 2). The graphs for the distribution of particle size of formulated CNPs are presented in Figure 1. As seen from TEM images in Figure 2, the NPs were spherical in shape.

### 3.2. NPs Stability under Variable Storage Condition

**Figure 3:**
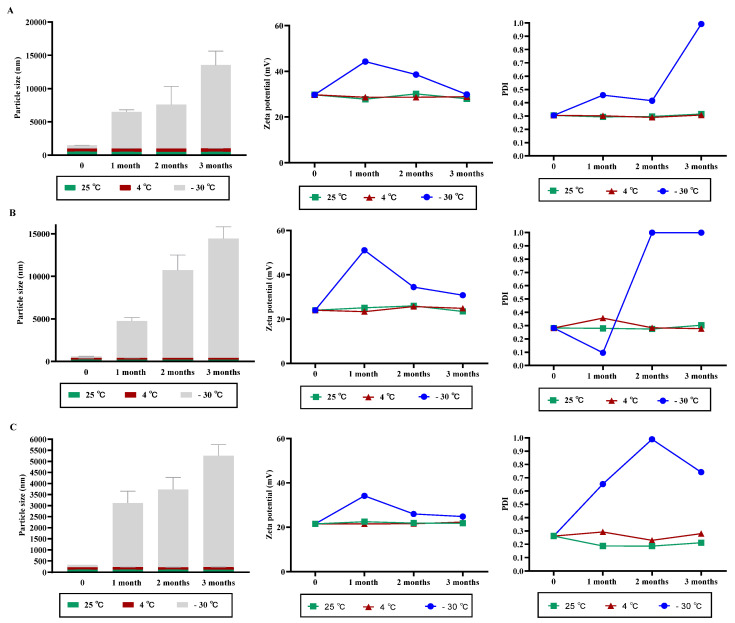
Characterization of CNPs at different time and storage conditions. Influence of different temperatures at different time points on the particle size, PDI, and ζ-potential of CNPs_5 mg/mL_ (**A**), CNPs_2.5 mg/mL_ (**B**) and CNPs_1 mg/mL_ (**C**) were evaluated. The data is presented as the mean ± SD of triplicate experiments.

As shown in Figure 3, no significant change in the particle size, PDI, and ζ-potential of CNPs was observed when stored at 4 °C for different time intervals as compared with fresh formulations. In contrast, storing NPs at −30 °C increased particle size significantly with an increase in storage intervals. Also, the PDI and ζ-potential of CNPs kept at −30 °C fluctuated markedly when compared with freshly prepared NPs (Figure 3A–C).

### 3.3. Antimicrobial Activity of CNPs

**Figure 4:**
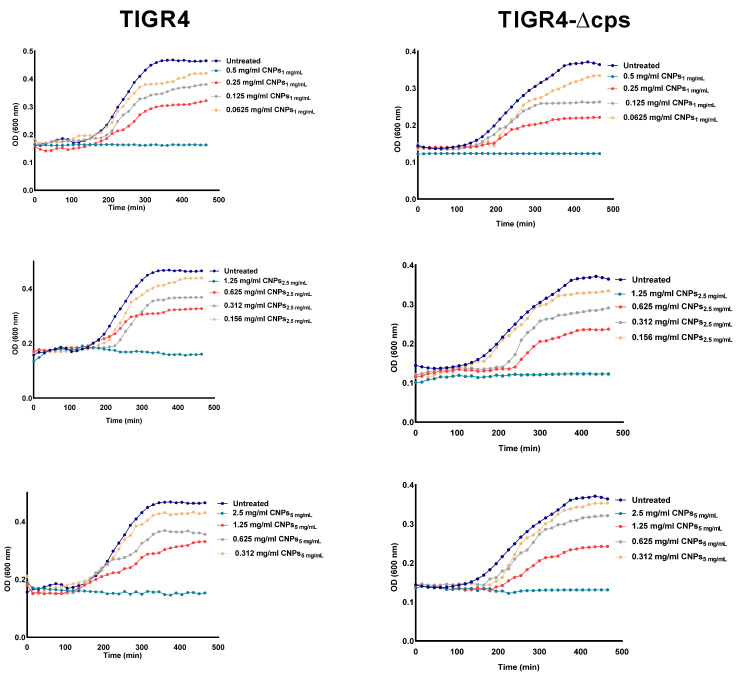
Growth curve of pneumococcal strains in the presence of CNPs. *S. pneumoniae* TIGR4 and TIGR4 ∆cps were cultured in THYB medium containing different concentrations of CNPs_1 mg/mL_, CNPs_2.5 mg/mL_, and CNPs_5 mg/mL_. Reduction in bacterial growth in the presence of CNPs was observed. The experiment was performed in triplicate. The data show one representative experiment.

The antimicrobial activity of prepared NPs was next assessed against *S. pneumoniae* strain TIGR4 and its capsule-negative mutant (TIGR4-∆cps.) using the broth dilution method. Initially, we tried to culture the pneumococcal strains at pH = 5.5, in which Cs is known to be protonated; however, bacteria did not grow under these conditions. Therefore, the experiment was performed at neutral pH. The optical density of the bacteria cultured in the presence of CNPs was subtracted from the optical density of the formulations alone. Growth curve results showed that although growth-inhibitory activity was observed at lower concentrations, the MIC_90_ was obtained at 0.5 mg/mL, 1.25 mg/mL, and 2.5 mg/mL for CNPs_1 mg/mL_, CNPs_2.5 mg/mL_, and CNPs_5 mg/mL_, respectively (Figure 4). The anti-bacterial effect of CNPs was not affected by the presence or absence of pneumococcal capsular polysaccharides (Figure 4).

**Figure 5:**
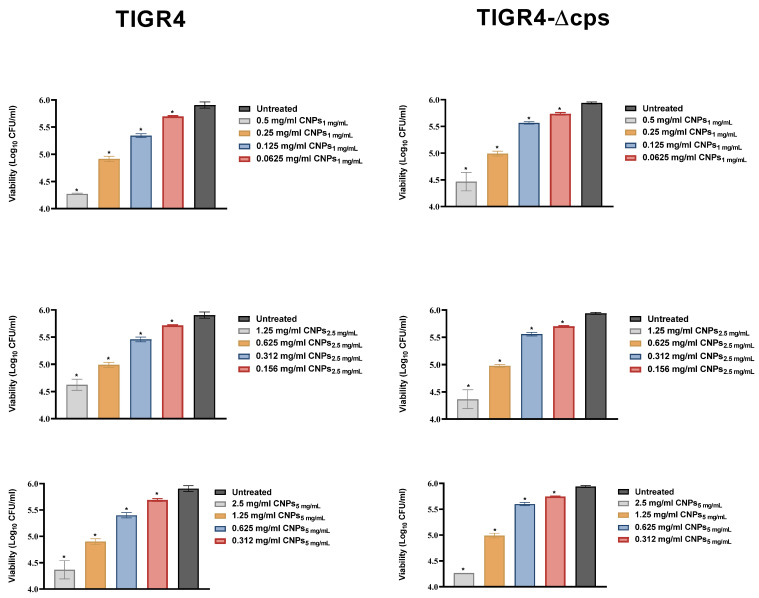
Effect of CNPs on bacterial killing. *S. pneumoniae* TIGR4 and TIGR4-∆cps were co-incubated with CNPs for 5 h, then serially diluted and plated. The number of colonies (CFU) were counted after overnight incubation. The data is presented as the mean ± SD of triplicate for one representative experiment. * *p* < 0.05 in ANOVA test relative to viability of untreated bacteria.

At higher concentrations of the formulations, the turbidity of the formulations in the media masked the turbidity resulting from bacterial growth; thus, a direct bactericidal assay was further conducted (Figure 5). As demonstrated in Figure 5, a dose-dependent reduction in bacterial viability was observed for the first four dilutions of each formula tested. Of note, the pH of the medium at first dilution in broth was approximately six.

### 3.4. The CNPs Reduced Pneumococcal Hemolysis Activity

**Figure 6:**
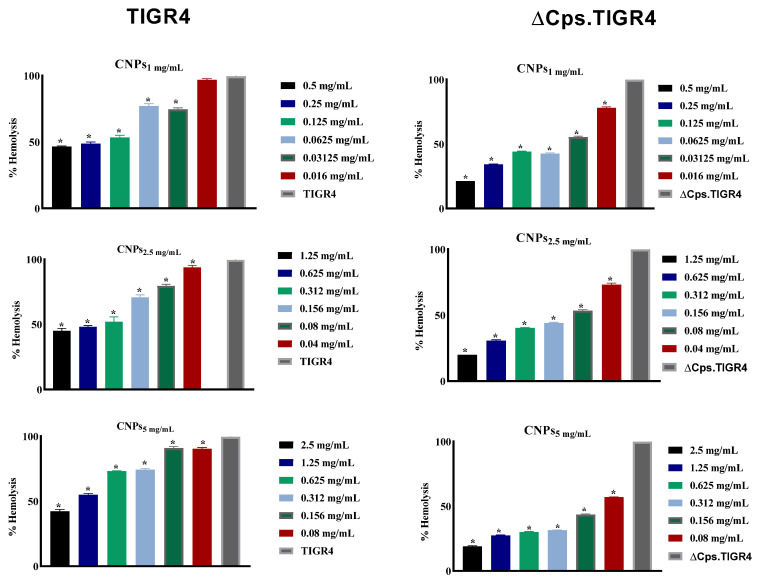
Effect of CNPs on the hemolytic activity of pneumococcal isolates. S. pneumonia TIGR4 and TIGR4-∆cps were co-incubated with CNPs for 5 h, bacterial supernatant was incubated with blood for 30 min, then absorbance was measured after centrifugation. The data is presented as the mean ± SD of triplicate experiments. * *p* < 0.05 in ANVOA test relative to hemolysis control of untreated bacterial supernatant.

The effect of CNPs on the hemolysis activity of bacterial culture supernatants was investigated using a hemolysis test. As observed in Figure 6, the CNPs induced a significant (*p* < 0.05), concentration-dependent reduction in the hemolytic activity of supernatant from TIGR4 and its capsule mutant. This suggests that CNPs inhibit bacterially induced hemolysis from pneumococcal supernatant. This is likely due to pneumolysin; however, this cannot be determined solely by the results of this study.

### 3.5. Evaluation of CNPs Cytotoxicity

**Figure 7:**
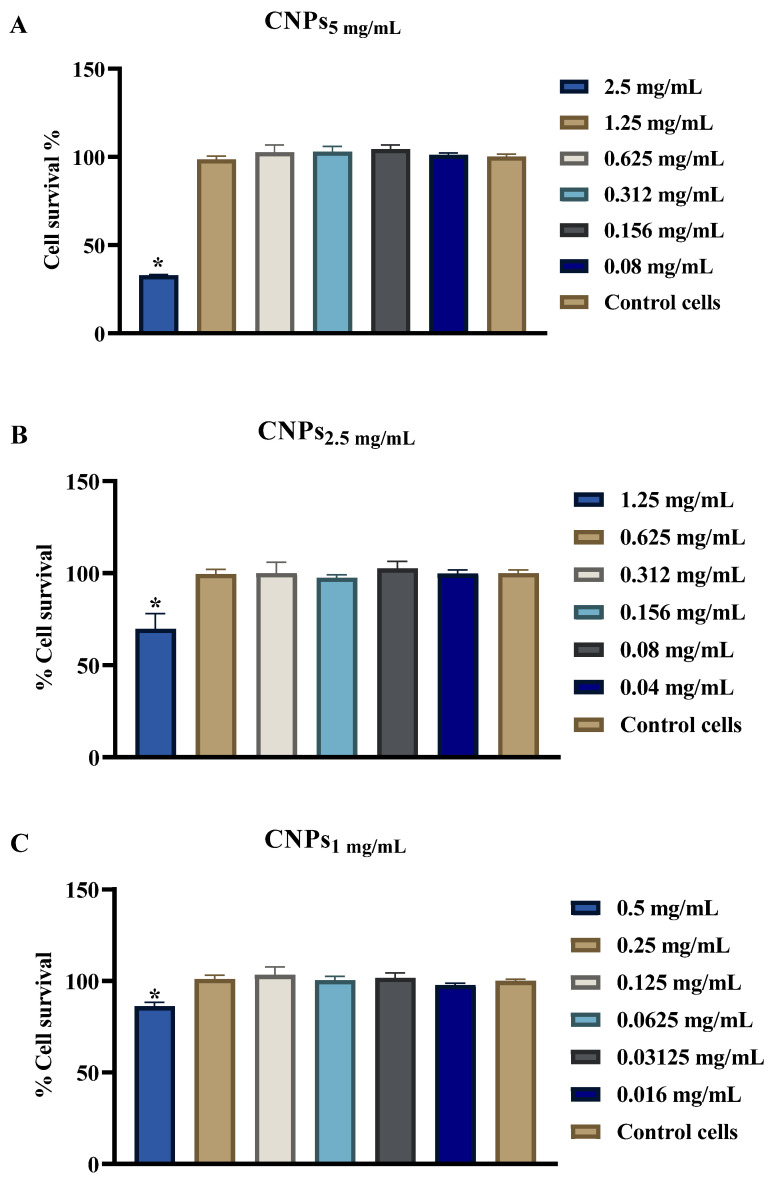
Effects of CNPs on A549 cell viability. Cells were left untreated or treated with different concentrations of CNPs_5 mg/mL_ (**A**), CNPs_2.5 mg/mL_ (**B**) and CNPs_1 mg/mL_ (**C**). Cell viability was assessed using alamarBlue assay. The data is presented as the mean ± SD of triplicate experiments. * *p* < 0.05 in Student’s *t*-test relative to control untreated cells.

In order to verify the safe use of CNPs as antimicrobials in humans, the cytotoxicity of synthesized NPs was studied on the A549 lung carcinoma host cell using the alamarBlue assay. The viability of cells exposed to different concentrations of CNPs is illustrated in Figure 7. A significant reduction in A549 cell viability was observed when treated for 24 hrs with 2.5 mg/mL concentrations of CNPs_5 mg/mL_ (Figure 7A). The CNPs_2.5 mg/mL_ and CNPs_1 mg/mL_ showed a less pronounced reduction in cell viability (30% and 23.8%, respectively at 1.25 mg/mL and 0.5 mg/mL concentrations; Figure 7B,C) and no cell death was observed at lower concentrations that still had antibacterial activity. Notably, synthesized NPs from CNPs1 mg/mL were the least cytotoxic nanoparticles at the MIC_90_ concentration utilized.

## 4. Discussion

The emergence of pneumococcal infections caused by multidrug-resistant strains represents a major global public health concern. In previous studies [12,13,14,15,16,17,18], Cs-based NPs have demonstrated a wide spectrum of antimicrobial properties. However, according to our knowledge, the current study is the first to highlight the potential use of CNPs against the clinical *S. pneumoniae* isolate TIGR4 and its capsule-negative mutant.

In the current investigation, we found that as the concentration of Chitosan was increased, the particle size and zeta potential of formed CNPs increased correspondingly in accordance with previous reports [12,16,18,25]. In the current study, we did not succeed in culturing pneumococcal isolates under acidic pH, which would provide optimal protonation of the chitin of the CNPs. Instead, the antimicrobial effect of CNPs was investigated under neutral pH. Using a standardized broth dilution assay, the MIC_90_ of CNPs_1 mg/mL_, CNPs_2.5 mg/mL_, and CNPs_5 mg/mL_ were found to be 0.5 mg/mL, 1.25 mg/mL, and 2.5 mg/mL, respectively. Cs anti-bacterial action is pH-dependent, and it is soluble in acidic environments, and thus, becomes polycationic when the pH is below the molecule’s pKa (6.3–6.5) [8,12]. Here, the inclusion of the CNPs in the assay media at these concentrations used shifted the pH to be slightly more acidic (approximately pH = 6), which is more optimal in providing protonation of the Chitosan. Several studies have reported antimicrobial activities of Cs and CNPs only at acidic pH [8,12]; however, this has not been proved to be strictly consistent. At lower pHs, Chitosan has stronger inhibitory activity that is reduced as pH rises. This explains the higher MIC90 results observed in our study as the pH of the media used for the assays was not acidic, resulting in the presence of a significant number of uncharged amine groups and low solubility of Cs at these conditions. There are several proposed mechanisms of antimicrobial action of Cs [12,26]. One mechanism depends on electrostatic interaction between negatively charged microbial membrane and positively charged Cs resulting in leakage of intracellular components. At pH = 7, the free Cs and outer Cs in prepared NPs are presumed to be neutral; however, the Cs charge within the NPs might be retained. Such retained positive charge might contribute to the antibacterial activity exhibited by CNPs synthesized in our study against pneumococcal isolates and other reports against *Streptococcus mutans* [17] and *E. coli* [27]. This might occur via the interaction of CNPs with negatively charged components, including peptidoglycan and teichoic acid in the cell walls of gram-positive bacteria. Another possible mechanism is the capacity of Cs to chelate metal ions in bacteria which has been reported for both acidic and neutral conditions [7,8]. Of note, Cs-mediated chelation of the metal ions is more effective at higher pH [8].

Capsular polysaccharide plays a significant role in pneumococcal survival to protect the bacteria from the host inflammation [27,28]. It contributes to pneumococcal virulence and is used for serotypes classification [29,30,31]. In our study, TIGR4 lacking capsular polysaccharide was found to be equally susceptible to the effect of CNPs as the wild-type strain. This finding suggests that the capsule material is not the target for CNPs and also that capsule does not interfere with CNP-induced anti-pneumococcal activity. It also might suggest that the contribution of other bacterial compartments, such as the cell wall or bacterial cell membrane in CNPs mediated anti-pneumococcal activity. The mechanism of CNPs bactericidal activity and specific targets will be addressed in a separate study.

Besides their direct bactericidal activities, the CNPs used here also displayed inhibition of hemolysis induced by pneumococci. We were able to demonstrate that the CNPs resulted in a concentration-dependent inhibition of pneumococcal hemolysis induced by TIGR4 and its capsule-negative mutant. This implies a potential anti-virulence effect of CNPs toward *S. pneumoniae*; however, the exact mechanism of the observed hemolysis attenuation is unknown from current findings. As pneumolysin is a known cholesterol-dependent toxin with hemolytic ability, it is tempting to speculate that CNPs may directly interact with pneumolysin and interfere with its function.

Finally, to provide evidence that CNPs may potentially be used safely to treat infections, the cytotoxicity of the CNPs prepared in this study was investigated against A459 lung carcinoma cells. Higher concentrations of CNPs_5 mg/mL_ were found to be markedly cytotoxic, but this was not observed for CNPs_2.5 mg/mL_ and CNPs_1 mg/mL_, suggesting that these formulations would be more favorable to use as therapeutics. Such cytotoxicity of CNPs prepared from higher concentrations of Cs is expected owing to their high zeta potential. These results corroborate other studies in which the CNPs cytotoxicity was evaluated on other cell lines such as HeLa cells [18].

## 5. Conclusions

In summary, the findings of this study reveal that the formulated CNPs produced capsule-independent anti-pneumococcal activity. NPs fabricated from lower concentrations of Cs were cytocompatible. In addition, the CNPs attenuate pneumococcal hemolysis in a concentration-dependent manner. As a result, the findings reported here suggest the potential of using nanochitosan as a future antimicrobial and anti-virulence strategy against *S. pneumoniae*.

## Figures and Tables

**Table 1:** Pneumococcal strains used in the current study.

*S. pneumonia* Strains	Description	References
TIGR4	*S. pneumoniae* serotype 4 clinical isolate	[20]
TIGR4-∆cps	Capsule-free TIGR4, Erm^R^	[21]

**Table 2:** Physicochemical characteristics of formulated CNPs.

Formulation	Size (nm ± SD)	PDI ± SD	ζ-Potential (mV ± SD)
CNPs_1 mg/ml_	133.3 ± 0.57	0.159 ± 0.006	19 ± 0.115
CNPs_2.5 mg/ml_	177 ± 2.85	0.235 ± 0.011	24.7 ± 1.06
CNPs_5 mg/ml_	423 ± 12.93	0.281 ± 0.014	27 ± 0.819
